# Molecular characterization of Gleason patterns 3 and 4 prostate cancer using reverse Warburg effect-associated genes

**DOI:** 10.1186/s40170-016-0149-5

**Published:** 2016-05-05

**Authors:** Ilinca Georgescu, Robert J. Gooding, R. Christopher Doiron, Andrew Day, Shamini Selvarajah, Chris Davidson, David M. Berman, Paul C. Park

**Affiliations:** Department of Pathology and Molecular Medicine, Queen’s University, Kingston, ON Canada; Division of Cancer Biology and Genetics, Cancer Research Institute, Queen’s University, Kingston, ON Canada; Department of Physics, Engineering Physics and Astronomy, Queen’s University, Kingston, ON Canada; Department of Urology, Queen’s University, Kingston, ON Canada; NCIC Clinical Trials Group, Queen’s University, Kingston, ON Canada; Ontario Institute for Cancer Research, Toronto, ON Canada

**Keywords:** Prostate cancer, Gleason pattern, Reverse Warburg effect, Biomarkers, Gene expression, NanoString

## Abstract

**Background:**

Gleason scores (GS) 3+3 and 3+4 prostate cancers (PCa) differ greatly in their clinical courses, with Gleason pattern (GP) 4 representing a major independent risk factor for cancer progression. However, Gleason grade is not reliably ascertained by diagnostic biopsy, largely due to sampling inadequacies, subjectivity in the Gleason grading procedure, and a lack of more objective biomarker assays to stratify prostate cancer aggressiveness. In most aggressive cancer types, the tumor microenvironment exhibits a reciprocal pro-tumorigenic metabolic phenotype consistent with the reverse Warburg effect (RWE). The RWE can be viewed as a physiologic response to the epithelial phenotype that is independent of both the epithelial genotype and of direct tumor sampling. We hypothesize that differential expression of RWE-associated genes can be used to classify Gleason pattern, distinguishing GP3 from GP4 PCa foci.

**Methods:**

Gene expression profiling was conducted on RNA extracted from laser-capture microdissected stromal tissue surrounding 20 GP3 and 21 GP4 cancer foci from PCa patients with GS 3+3 and GS ≥4+3, respectively. Genes were probed using a 102-gene NanoString probe set targeted towards biological processes associated with the RWE. Differentially expressed genes were identified from normalized data by univariate analysis. A top-scoring pair (TSP) analysis was completed on raw gene expression values. Genes were analyzed for enriched Gene Ontology (GO) biological processes and protein-protein interactions using STRING and GeneMANIA.

**Results:**

Univariate analysis identified nine genes (*FOXO1* (*AUC: 0.884*), *GPD2*, *SPARC*, *HK2*, *COL1A2*, *ALDOA*, *MCT4*, *NRF2*, and *ATG5*) that were differentially expressed between GP3 and GP4 stroma (*p*<0.05). However, following correction for false discovery, only *FOXO1* retained statistical significance at *q*<0.05. The TSP analysis identified a significant gene pair, namely *ATG5/GLUT1*. Greater expression of *ATG5* relative to *GLUT1* correctly classified 77.4 % of GP3/GP4 samples. Enrichment for GO-biological processes revealed that catabolic glucose processes and oxidative stress response pathways were strongly associated with GP3 foci but not GP4. *FOXO1* was identified as being a primary nodal protein.

**Conclusions:**

We report that RWE-associated genes can be used to distinguish between GP3 and GP4 prostate cancers. Moreover, we find that the RWE response is downregulated in the stroma surrounding GP4, possibly via modulation of *FOXO1*.

**Electronic supplementary material:**

The online version of this article (doi:10.1186/s40170-016-0149-5) contains supplementary material, which is available to authorized users.

## Background

In order to assess the risk of metastasis in prostate cancer (PCa), Gleason grading integrates the relative abundance of cancer cells that make low-grade patterns (Gleason pattern 3 or GP3) with those that make high-grade patterns (Gleason patterns 4 or 5 or GP4 and GP5). Cancers with more abundant high-grade patterns obtain higher Gleason scores (GS) and have higher risk of metastasis and death. Those with only low-grade cancer cells have almost no risk of PCa-specific death [1, 2]. GS exhibits a strong correlation with clinical outcome. However, scores are often misreported due to differences in grading performed by individual pathologists, and under-sampling during biopsy, which occurs in up to 30 % of cases [3]. Prognostic biomarkers are urgently needed to augment clinicopathologic parameters, such as GS, in the risk stratification of this disease. Unlike other epithelial tumors, such as breast tumors, PCa lacks confirmed molecular subtypes that differ in their prognosis or treatment response. Several biomarkers/panels that have been identified to date have thus far not been widely embraced by clinicians [4–11]. Notably, the gene features among these panels exhibit little overlap, reinforcing the notion of molecular heterogeneity in the progression of PCa [12–15].

Emerging evidence indicates that in many cancers, the tumor microenvironment plays a crucial role in their phenotypic progression [16–19]. Data from cell lines and animal models indicate that reciprocal interactions between cancer cells and cancer-associated fibroblasts (CAFs) facilitate growth and dissemination of tumor cells [20–24]. Central to this reciprocal relationship is the paradigm of altered glucose metabolism and metabolic coupling between cancer cells and CAFs known as the reverse Warburg effect (RWE) [25]. Specifically, cancer cells induce oxidative stress in adjacent stroma by promoting autophagy and lysosomal destruction of the mitochondria in CAFs, thereby diverting their favored metabolic pathway to aerobic glycolysis. The resulting high-energy by-products, such as lactate and ketones, are shuttled back to the tumor cells, in which they fuel increased oxidative phosphorylation and efficient ATP production needed for anabolic growth [26]. Oxidative stress also enhances the production of free radicals, resulting in increased DNA damage and random mutagenesis in cancer cells [27, 28]. This relationship is maintained in most aggressive cancers and is indeed recognized as one of its emerging hallmarks.

Given that the changes experienced by the stroma are likely to be reflective of cancer growth and progression, probing the metabolic state of CAFs may provide a means of indirectly assessing the phenotypic state of the cancer cells, while bypassing the problem of epithelial genotypic heterogeneity and the need for direct sampling of the cancer cells themselves [29, 30]. Additionally, because the tumor microenvironment is comprised of relatively benign cells, its genomic features are likely to be more reliable [31–34], making it an ideal medium in which to profile physiological responses, such as the RWE, to genetically dissimilar patterns of PCa.

Therefore, in this study, we compare the RWE status in the stromal component between aggressive and indolent PCa foci. Specifically, we compare the expression levels of 102 RWE-associated genes in the stroma adjacent to GP3 foci from GS 3+3 tumors to those of GP4 foci from GS ≥4+3 tumors. We report two potential classifiers that discriminate GP3 from GP4 tumor foci. Based on the clinical behavior and the histologic features of GS 3+3 and GS >4+3 tumors, we propose potential roles for these genes in establishing their respective phenotypes.

## Methods

### Human prostate tumor samples

This study was conducted under the approval of the Research Ethics Board at Queen’s University. The pathology database at the Kingston General Hospital was queried for radical prostatectomies between the years 2001 and 2013. Through the review of the pathology reports, suitable cases were identified for the two groups in our cohort. The first consisted of cases of organ-confined diseases of GS 3+3, with no evidence of higher tertiary pattern. The second group consisted of diseases with GS ≥4+3, with or without evidence of extraprostatic involvement (Additional file 1: Table S1). Together, the GS 3+3 and the GS ≥4+3 groups represent divergent prognoses, “low-risk” and “intermediate to high-risk,” respectively, for localized PCa [35]. Each of the selected cases were retrieved for histologic review by one of two urologic pathologists (DMB, CD) to confirm the diagnosis, according to the International Society of Urological Pathology (2005) Consensus guidelines [36]. In total, 20 GS 3+3 samples and 21 GS ≥4+3 samples were selected for this study.

### Sample processing

GP3 foci and GP4 foci were identified from hemotoxylin and eosin-stained slides of GS 3+3 and GS >4+3 samples, respectively. Corresponding archival formalin-fixed paraffin-embedded (FFPE) blocks were sectioned and mounted on slides. Stromal tissue adjacent to cancer foci was harvested by laser capture microdissection, using a Zeiss PALM CombiSystem microscope. A minimum of 3 ×106 *μ*m^2^ of stroma was harvested for each sample, using multiple serial sections, where necessary, to restrict the field of harvest to within ten cell widths from the margin of the epithelial foci.

RNA was extracted from microdissected tissue using the RNeasy ^Ⓒ^ FFPE Kit (Qiagen, Valencia, CA, USA). The manufacturer’s protocol was modified to substitute the proteinase digestion with that of Roche’s PCR-grade recombinant Proteinase K (Roche Diagnostics Mannheim, Germany) at 56 °C for 30 min (18.6 mg/ml). The final elution step was conducted using RNase-free water heated to 37 °C and repeated twice in order to increase yield. RNA was quantified using the Agilent RNA 6000 Pico Kit (Agilent Technologies, Santa Clara, CA, USA), following manufacturer’s protocol, and stored at −80 °C until use. RNA quality was assessed by smear analysis and RNA integrity numbers (RIN) using the Agilent 2100 Bioanalyzer.

### Compilation of candidate RWE gene panel

A gene panel representative of the RWE was generated using a three-pronged in silico approach. As a starting point, the literature was mined to identify genes with known associations to the RWE in both breast and prostate carcinomas [25, 27, 28, 37–42]. By design, genes from the primary list were grouped based on primary biological function into the following categories: hypoxia response/oxidative stress regulation, mitophagy, autophagy and mitochondrial dysfunction, glucose metabolism, myo-fibroblast differentiation and CAF markers, and metabolite transporters. These small groups of genes were then input into a network-building algorithm, STRING (http://string-db.org), and nodal points that possessed a combined functional-evidence confidence score of greater than 0.9, with more than three of the input genes, were noted.

Lastly, in order to further enrich the target gene list, the following Gene Omnibus (http://geneontology.org) [43] databases comparing the transcriptomes of laser-captured microdissected or cultured stromal tissue derived from normal and invasive human breast and prostate carcinomas were accessed: GSE34312 [44], GSE26910 [45], and GSE11682 [46]. For each dataset, samples were assigned to either “normal-associated” or “tumor-associated” groups based on experimental labeling and compared using the GEO2R analysis software provided by the Gene Omnibus database (http://www.ncbi.nlm.nih.gov/geo/geo2r/). GEO2R generates a list of the top 250 differentially expressed genes, ranked by *p* value. Genes with an adjusted *p*<0.05, an absolute value of the log base two fold change >1.5, and a biological function that fits into either of the above-mentioned categories were marked for inclusion. Genes identified through network building and Gene Omnibus database searches were subject to literature review prior to inclusion in the final gene panel. The final panel consisted of 102 target genes (Additional file 2: Table S2) associated with RWE for use in gene expression profiling. Five housekeeping genes were selected for inclusion, namely *ACTB, CLTC, GUSB, HPRT1*, and *TUBB*, as these genes had proven suitable for normalization in previous PCa gene expression profiling studies [47].

### cDNA conversion and multiplexed target enrichment for nCounter analysis

Prior to hybridization, target enrichment was performed using a multiplexed target enrichment (MTE) protocol (NanoString Technologies, Seattle, WA, USA). Primer pairs were designed for each of the 102 target genes using Primer3 software. These primer pairs (Integrated DNA Technologies, Coralville, IA, USA) flanked the 100-nucleotide target regions specific to the NanoString probes (Additional file 3: Table S3). Two nanograms of RNA for each sample was reverse transcribed and amplified for 20 cycles, using SuperScript VILO MasterMix (Life Technologies, Carlsbad, CA, USA) and TaqMan ^Ⓒ^ PreAmp Master Mixes (Applied Biosystems, Foster City, CA, USA), respectively.

### Sample hybridization and nCounter analysis

A digital multiplexed NanoString nCounter analysis (NanoString Technologies, Seattle, WA, USA) for gene expression was performed using 11 *μ*L of denatured amplified stromal cDNA. Each sample was probed against the custom RWE NanoString panel, which included 102 RWE-associated genes, 5 housekeeping genes, 6 spiked-in positive controls ranging in concentration from 128 to 0.128 fM, and 8 synthetic negative control sequences (Additional file 4: Table S4). The hybridization reaction was prepared according to the nCounter Single Cell Expression Assay protocol (NanoString Technologies, Seattle, WA, USA). The digital analyzer pre-processed barcode images internally according to standard specifications for binding density and field of view (FOV). All samples were used, as their binding density was inside the recommended 0.05–2.25-range, and their percent FOV was greater than 75/280.

### Processing and data normalization

A protocol for NanoString gene expression data normalization was developed in-house based on the NanoStringNorm Bioconductor package [48]. Data normalization included positive control normalization, background correction, and sample content normalization Additional file 5: Figure S1 and Additional file 6: Figure S2. Our housekeeping collection was not used in normalization since only one gene, *ACTB*, showed reproducibility across all samples Additional file 7: Figure S3. (Within the NanoStringNorm package, the relevant options were CodeCount = “geo.mean,” Background = “mean,” SampleContent = “top.geo.mean.”)

Briefly, the geometric mean of the six spiked-in positive controls taken across all lanes was divided by the geometric mean of each lane to create positive control correction factors for each sample. Positive normalization factors were accepted if they fell within the recommended range of 0.3–3. Following positive control normalization, the mean of the negative control counts was used to estimate the background for each lane. This less conservative approach allowed for low expression profile distributions to be statistically probed. The sum of the geometric means for the top 75 genes with the highest intensities was divided by the sum of the top 75 genes within a single sample in order to produce a sample content normalization factor for each lane. The sample content correction factors were then multiplied against the control-normalized data. Sample content normalization factors were deemed acceptable if they ranged between the standard 0.1 and 10. Prior to our final statistical analysis, those samples with >50 % missing data were excluded. Similarly, genes with >50 % missing data across samples were discarded.

### Statistical analysis

Univariate gene expression differences were assessed using the rank-based non-parametric Mann-Whitney *U* (MWU) test, appropriate for manifestly non-normal distributions, as well as the Welch *t* test, noting the nonequivalence of sample variances. To account for small sample numbers, we employed both tests with a cutoff of *p*=0.05, being more confident in identifying expression profile differences if both the medians and means were found to be different. The Benjamini-Hochberg false discovery rate (FDR) correction [49], using the *p.adjust* routine from R, was applied to both *p* values to account for multiple comparisons, thereby producing a *q* value for each test. Receiver operator curves (ROC) were generated for genes of interest in order to determine their accuracy in distinguishing GP3 from GP4.

Top-scoring pair (TSP) analysis [50] was used to identify pairs of genes that successfully classify GP3 from GP4. This method is employed to find a classifier that is not dependent on certain subjective decisions, specifically the calculation of sample content normalizations, or the setting of arbitrary cutoffs for normalization factors made when processing and normalizing the data, or the expression of reference genes. Here, prior to TSP analysis, raw data were mean-background corrected, followed by the exclusion of genes and then samples possessing >50 % zero values. Permutation testing was used to query the significance of the top-scoring pair under the null hypothesis that gene count is not associated with Gleason pattern. One hundred thousand random classification assignments were run to generate a score distribution.

### Pathway analysis

Pathway analysis was conducted using STRING (http://string-db.org) [51] and GeneMANIA (http://www.genemania.org) [52]. All genes that exhibited an area under the curve (AUC) greater than 0.7 in the ROC analysis were used as input. The gene list was then augmented with those having a *p*<0.1 for either test with a minimum fold change of 1. In total, 17 upregulated genes and 4 downregulated genes, in GP3 relative to GP4, were used as inputs. Input genes were enriched for 5 and 10 related genes in STRING. Network significance, in all cases, was based on a GO [53] biological process-based weighting (http://geneontology.org) with correction for FDR. The STRING results were compared to those found using GeneMANIA software.

## Results

### Assessing stromal FFPE sample quality

All RNA samples had RINs ranging between 2 and 2.5, typical of fragmented RNA extracted from FFPE tissue [54]. Smear analysis revealed variability in the amount of fragmentation between samples, with the percentage of 50–300 residue length fragments ranging between 50 and 84 %. Low yields and fragmentation of RNA necessitated RNA amplification prior to profiling.

### Processing and normalization of NanoString gene expression data

All 41 samples passed binding density and FOV quality control measures implemented by NanoString. One sample (GP4-18) was removed during QC assessment due to its large normalization factor. Even with the application of the modestly conservative background correction, low counts resulted in a significant proportion of zero values within samples, as well as across samples for select genes. To eliminate unreliable data, seven samples which failed to register counts in >50 % of the genes (GP3-5, GP3-6, GP3-7, GP3-12, GP3-13, GP3-20, and GP4-12) were excluded from further analysis. Similarly, two genes (*NOS2* and *TKTL1*) which failed to register counts in >50 % of the samples were also excluded from further analysis. Following data normalization and exclusion based on missing values, 98 genes and 33 PCa samples (15 GP3, 18 GP4) passed all thresholds set for normalization and background correction and were included in downstream univariate analysis.

### Univariate analysis of differentially expressed genes associated with RWE

Univariate statistical analysis was applied to normalized gene expression data, followed by in-house data processing techniques. In the present cohort, nine genes were differentially expressed between GP3 and GP4 stroma using both the MWU and Welch *t* tests (*p*=0.05). These genes (Table 1), listed in order of statistical significance, are *FOXO1, GPD2, SPARC, HK2, COL1A2, ALDOA, SLC16A4 (MCT4), NRF2*, and *ATG5*. Two additional genes, *SIRT3* and *ACTA2*, were found to be significant by the Welch *t* test but not by the MWU test. Seven of differentially expressed genes, *FOXO1 GPD2, HK2, ALDOA, SLC16A4, ATG5*, and *SIRT3*, were upregulated in the stroma from GP3 relative to GP4, while *SPARC, COL1A2, NRF2*, and *ACTA2* were downregulated in GP3 stroma relative to GP4. Notably, *FOXO1, SPARC, HK2*, and *MCT4* exhibited the highest magnitude of log_2_ fold changes of 4.58, 3.12, 3.81, and 3.46, respectively.

**Table 1:** Differential expression of RWE-associated genes in GP3 versus GP4 stroma

Gene	*p* (Welch *t*)	*p* (MWU)	*q*	ROC (AUC)	Log_2_ ($\overline {\mathrm {GP4}}/\overline {\mathrm {GP3}}$)
*FOXO1*	0.0008	0.0005	0.0495	0.884	–4.590
*GPD2*	0.0046	0.0034	–	0.707	–3.58
*SPARC*	0.0194	0.0119	–	0.769	3.12
*HK2*	0.0172	0.0132	–	0.752	–3.82
*COL1A2*	0.0191	0.0200	–	0.742	2.16
*ALDOA*	0.0118	0.0231	–	0.778	–2.60
*SLC16A4*	0.0392	0.0327	–	0.750	–3.43
*NRF2*	0.0197	0.0349	–	0.729	2.46
*ATG5*	0.0239	0.0410	–	0.748	–2.79
*ACTA2*	0.0255	0.1083	–	–	0.90
*SIRT3*	0.0375	0.1505	–	–	–3.25

**Fig. 1:**
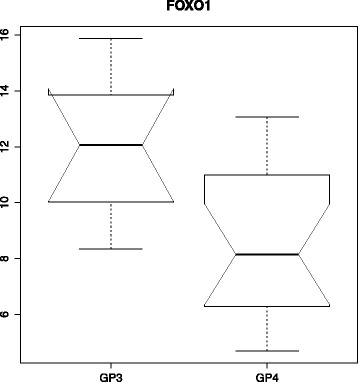
Boxplot of GP3 vs. GP4 expression for *FOXO1*. A notched box-and-whisker plot representing the distributions in *FOXO1* gene expression between GP3 and GP4. (The *solid lines* represent the medians, and the notches show the 95 % confidence intervals for the medians. The *whiskers* represent 1.5 times the inter-quartile ranges.) The *y*-axis represents log _2_ expression intensities. The upper GP3 quartile and lower GP4 quartile show minimal overlap, and the median show significant separation, consistent with the results of the MWU test. *FOXO1* retained statistical significance even after FDR was applied to the MWU test (*q*=0.0495)

To account for multiple comparisons, a Benjamini-Hochberg FDR correction was applied to the MWU test values using *p*.*adjust* from the Bioconductor R package. As expected, the FDR was very high given the small sample size compared to the number of variables. After correction for FDR, only one gene, *FOXO1*, retained statistical significance with an adjusted *p* value of *q*=0.0495. A notched box-and-whisker plot for this gene’s expression values is shown in Fig. 1.

**Fig. 2:**
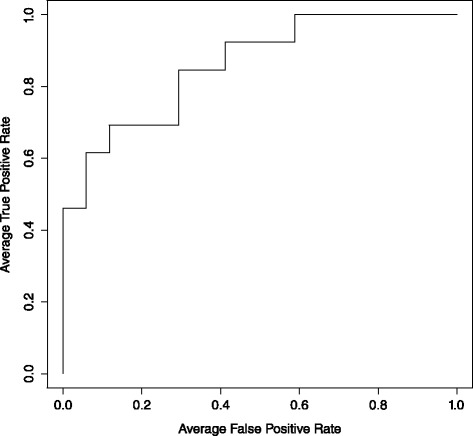
ROC curve for *FOXO1*. AUC =0.884

**Fig. 3:**
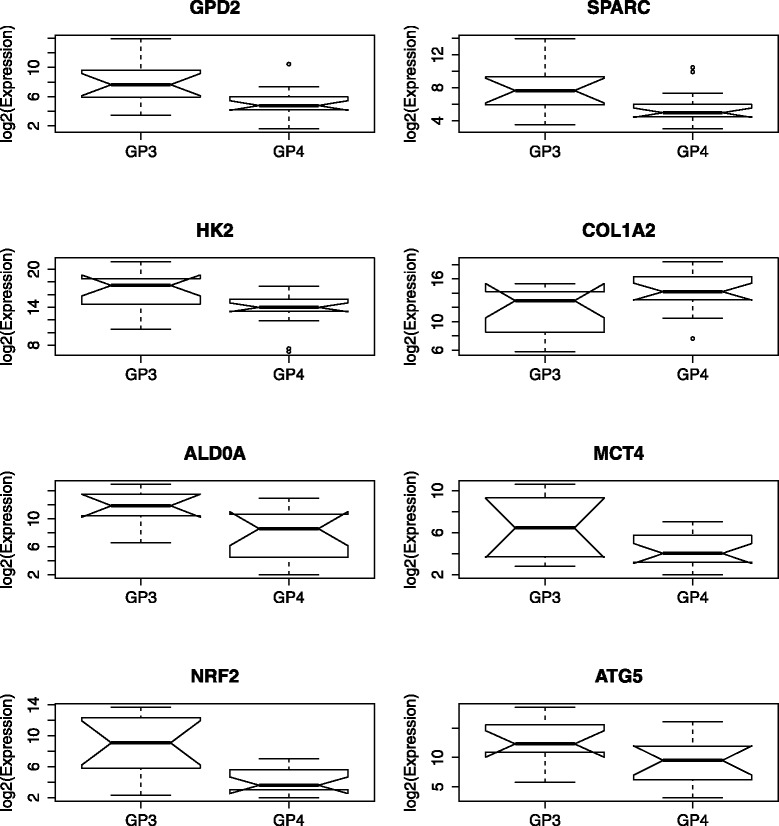
Boxplot of GP3 vs. GP4 expression for various genes. Notched boxplots for the eight genes, after *FOXO1*, which were found to be differentially expressed. For these genes, the *p* value found in both a parametric (Welch *t* test) and non-parametric (Mann-Whitney *U* test) were less than 0.05, indicating that both the means and medians differentiated between GP3 and GP4. The *symbols* and *notation* used in these plots are the same as that employed in Fig. 1

To assess the suitability of the differentially expressed genes as classifiers, ROC curves were generated. *FOXO1* produced an AUC of 0.884 (Fig. 2), suggesting a high discriminatory power between GP3 and GP4. The remaining eight genes had individual AUCs ranging between 0.71 and 0.78. Notched box-and-whisker plots for these eight genes are shown in Fig. 3.

### Pathway analysis

**Fig. 4:**
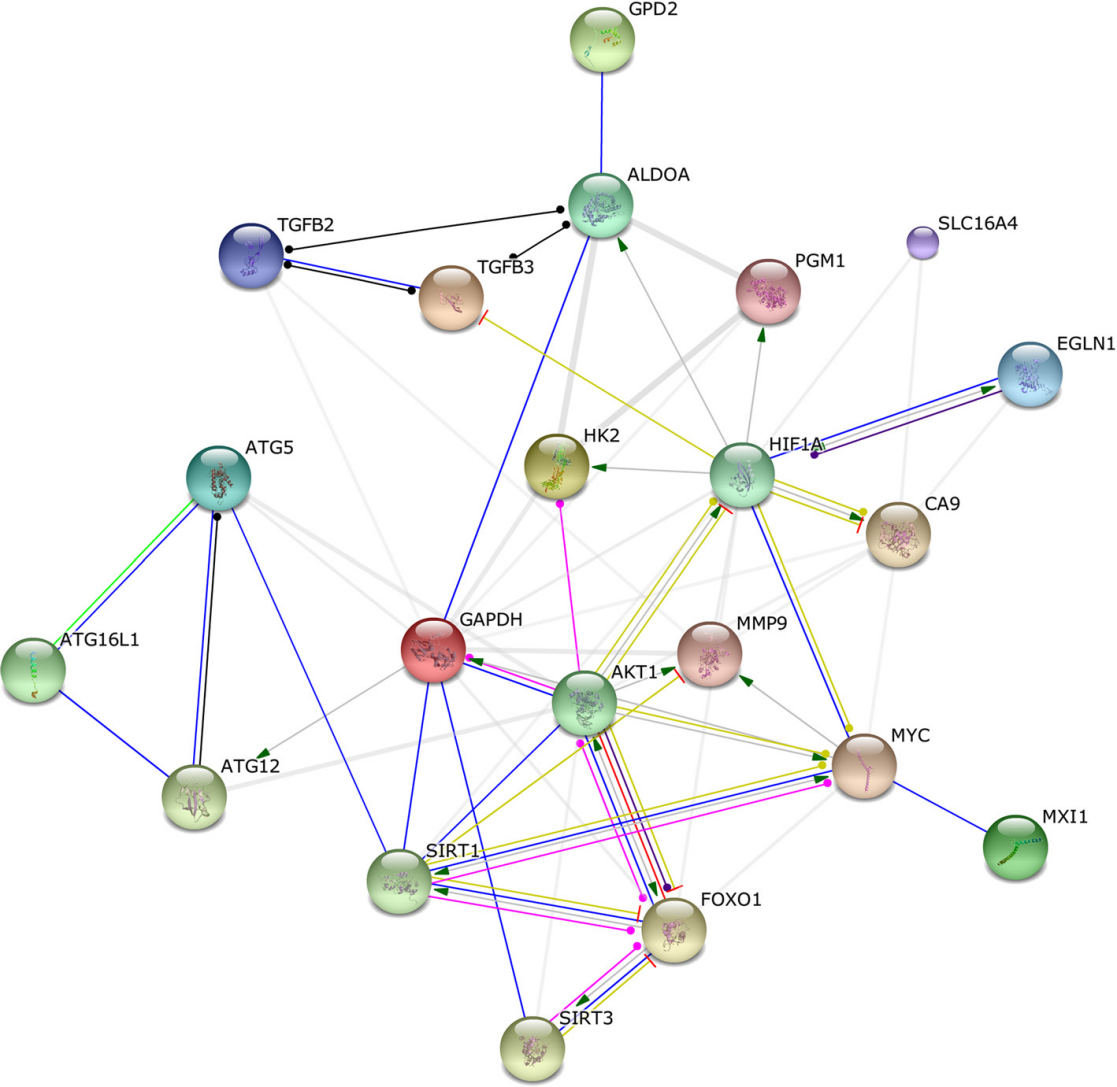
Protein-protein interactions network generated using STRING. The network is made up of the 17 upregulated genes in GP3 relative to GP4 (*FOXO1, GPD2, HK2, MYC, ALDOA, SLC16A4, ATG5, TGFB2, TGFB3, EGLN1, GAPDH, CA9, P4HA1, MXI1, MMP9*, and *PGM1*), plus five enriched genes (*ATG16L1, ATG12, AKT1, SIRT1*, and *SIRT3*). The network centers on the primary nodal points: *FOXO1, AKT1, MYC*, and *HIF1A*, which show the largest number of reciprocal inhibitory and activating functions with each other as well as with their interacting proteins. Activation (*green*), inhibition (*red*), binding (*blue*), post-translational modification (*fuchsia*), reaction (*black line*). Directionality is indicated by the *arrow*

To establish signalling networks that may play significant biological roles in GP3 and GP4, subject to the provision that genes are taken only from our original RWE gene list, STRING pathway analysis software was employed using enrichment for the most meaningful GO biological processes. Irrespective of the use of all 21 genes, namely 17 upregulated genes (*FOXO1, GPD2, HK2, MYC, ALDOA, MCT4, ATG5, TGFB2, TGFB3, EGLN1, GAPDH, CA9, P4HA1, MXI1, MMP9*, and *PGM1*) and 4 downregulated genes (*COL1A2, SPARC, NRF2*, and *TGFBR2*) or only those 17 genes that are upregulated in GP3 relative to GP4, three pathways were consistently identified: “gluconeogenesis,” “hexose catabolic process,” and “monosaccharide catabolic process” (Table 2). Analysis of the protein-protein interactions within the 26-gene network identifies *FOXO1* and *AKT1* as being primary nodal points based on their number of connecting proteins (Fig. 4). *AKT1* and *FOXO1* appear to directly affect one another through reciprocal activation or inhibition in a manner that is dependent on the overall metabolic context. Two processes that are not found when one uses only our input genes, but that are consistently reported upon enrichment of five or ten related genes, are the “response to oxygen levels” (*p*=2.97×10^−4^) and “response to hypoxia” (*p*=3.91×10^−4^). To validate these results, the same process was repeated in GeneMANIA with the addition of ten related gene partners. The prevalence of the glucose catabolic pathway was recurrent in both analyses, further substantiating this observation.

**Table 2:** Enrichment for GO biological processes using STRING

Term	Number	*p*	*q*
	of genes		
Gluconeogenesis	4	5.92×10^−8^	4.54×10^−4^
Hexose catabolic process	4	1.42×10^−7^	4.54×10^−4^
Monosaccharide catabolic process	4	2.33×10^−7^	4.86×10^−4^

### TSP analysis

In order to limit normalization biases resulting from the small number of samples, rank-based TSP of pre-processed raw data was also tested. However, the broad distribution of negative controls (Additional file 6: Figure S2) indicated that the low intensity readings in our raw dataset were unlikely to be reliable in its present form. Therefore, we chose to apply background correction using the built-in exogenous negative controls. Backgrounds were subtracted from the raw data using the modestly conservative “mean” option in the NanoStringNorm package.

Following mean background correction, and exclusion of genes and samples with >50 % missing data, four genes and nine samples were excluded. Excluded genes and excluded samples were as follows: *HGMCL, IGF2, IL10, NOS2*, and *TKTL1* and GP3-5, GP3-6, GP3-7, GP3-10, GP3-12, GP3-13, GP3-14, GP3-20, and GP4-12, respectively. Additionally, the single sample (GP4-18) that did not previously pass sample content normalization due to its large scaling factor was also removed, in order for our treatment of the data to remain consistent between the univariate and TSP analyses. The final dataset used for TSP analysis consisted of 13 GP3 and 18 GP4 samples (Additional file 7: Figure S3).

**Fig. 5:**
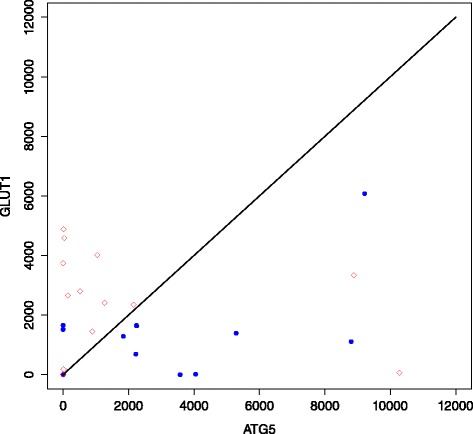
TSP scatterplot. Scatterplot illustrating the separation of GP3 and GP4 using the expression intensities of gene pair *ATG5/GLUT1*. With the ordering Exp(*ATG5*) > Exp(*GLUT1*), 24/31 (77.4 %) samples were correctly classified. One outlier (which agrees with the ordering) has been left out for clarity

**Fig. 6:**
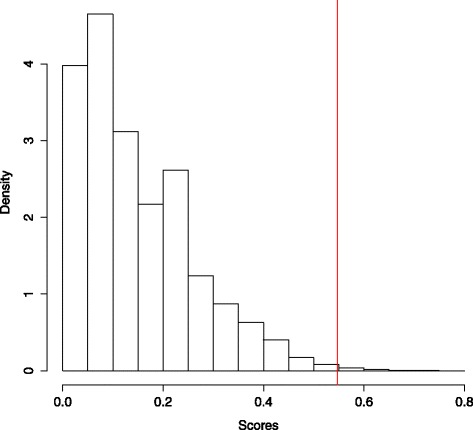
TSP random classification distribution. Distribution of 100,000 random classification assignment TSP samplings. The low frequency of high scores indicates that the score (0.547—*red line*) identified for top-scoring gene pair (*ATG5/GLUT1*) is unlikely to be due to chance (*p*=0.0039)

TSP analysis identified a top-scoring pair *ATG5/GLUT1* with a score of 0.547, capable of correctly classifying 24/31 (77.4 %) samples. To be specific, with the ordering (Exp(*ATG5*) > Exp(*GLUT1*)), the scatterplot of Fig. 5 was generated. Permutation testing using 100,000 random assignment classifications of GP3 and GP4 into groups of 13 and 18 generated a distribution of scores (Fig. 6). The low frequency of scores greater than 0.5 indicate that a score of 0.547 for gene pair *ATG5/GLUT1* is highly significant (*p*=0.0039).

## Discussion

The roles of the stromal microenvironment and the RWE have become increasingly noteworthy in the context of cancer progression, and, as such, suggest a potential utility of RWE-associated genes as prognostic biomarkers. Our study has identified an RWE-associated gene, *FOXO1* (AUC: 0.884), that is significantly differentially expressed between GP3 and GP4 stroma, even after FDR correction, as well as a multivariate top-scoring RWE-associated gene pair, *ATG5/GLUT1*, whose relative expression can classify GP in 77.4 % of cases. The remaining eight genes, which were significantly differentially expressed in both Welch and MWU tests, but did not reach statistical significance upon FDR correction, are also suggestive of additional classifiers.

If reproducible in subsequent independent cohorts, these RWE-associated biomarkers may have clinical value in risk stratification. The identification of *FOXO1* and *ATG5/GLUT1* in purified stroma and their differential expression in aggressive vs. indolent prostate cancer samples indicate that it is possible to clinically categorize PCa in terms of the metabolic responses, namely RWE, that it elicits in the stroma. Additionally, if these gene expression changes extend appreciably beyond the immediate tumor-stroma border, direct sampling of GP4 or GP3 may become less necessary. For example, decreased expression of *ATG5* relative to *GLUT1* in a biopsy core showing predominantly stroma or only low-grade cancer may indicate the presence of nearby higher-grade cancer that was missed by the biopsy. Evidence to suggest that this type of “field effect” exists within the stroma includes studies that show high autophagic turnover in fibroblasts at a distance of up to 5 mm away from the cancer [55].

In addition to the identification of two significant classifiers, this study has also provided potential insights into the RWE response as it pertains to different grades of PCa. The expression profiles of low-grade cancer stroma reported here are consistent with Pavlides’ model of the RWE, which depend on ROS-induced and *HIF1A*-mediated transcription of genes encoding key glycolytic enzymes, transporters, and autophagic vesicle assembly factors [35]. The upregulation of *MCT4* seen here is consistent with the induction of reciprocal lactate shuttling during RWE [56]. Similarly, the upregulation of genes, *HK2, ALDOA, GPD2*, and *ATG5*, which are prerequisites for responses to high glycolytic influx and auto-phagosome formation, respectively, are also consistent with the literature on RWE induction [25, 57]. Interestingly, both of the statistically meaningful classifiers, *FOXO1* and *ATG5*, are directly implicated in the activation of the mitochondrial autophagy response. More specifically, *FOXO1* promotes the transcription of the *ATG5* gene [58]. These congruent results, achieved via two different means of data processing, highlight an association between autophagy and GP3 and validate earlier studies that identify mitochondrial dysfunction as the primary mechanism of RWE induction in CAFs [40, 55]. Similarly, since *GLUT1* expression in stromal cells reflects their ability to import and metabolize glucose, the relative expression of the TSP genes *ATG5* >*GLUT1* may indicate that autophagy may be more important to RWE establishment and maintenance than upregulation of glucose intake.

It is, however, noteworthy that other common mitophagy markers such as *BNIP3* and *MAP1LC3B* were not found to be differentially expressed between the Gleason patterns. *ATG5* and *BNIP3* participate in early and late autophagy response, respectively [59], and therefore the upregulation of *ATG5* primarily points towards a role for early autophagosome formation in GP3-induced RWE. *HIF1A* also showed no significant difference in expression; however, upon enrichment for GO biological processes, responses to hypoxia and oxygen availability were revealed to be significant. Lastly, we did not observe expression differences in *LDHA* and *LDHB*, the enzymes involved in conversion of pyruvate to lactate, or in *PDK1* which regulates the flux into the tricarboxylic acid cycle. In our context, this may indicate that lactate production may not be as important as lactate export to the RWE in PCa.

Relative to stroma from low-grade cancer, higher-grade stroma exhibited gene expression patterns that indicated a reduced RWE response. These results are in agreement with studies conducted by Koukourakis et al. [60–62] and Rattigan et al. [63] who have reported that CAFs isolated from both lung and colorectal cancers retain their oxidative phosphorylation potential, likely in order to recycle the high volume of lactate secreted by more aggressive tumors. These results also corroborate studies conducted on breast and pancreatic cancers that have reported the reduced importance of RWE in more aggressive disease subtypes such as TNBC and HER2+ [64]. This reduction in RWE response in GP4 relative to GP3 foci leads us to speculate that RWE is perhaps a characteristic of a predominantly proliferative phenotype with rather than invasion and metastasis.

This switch from a predominantly proliferative phenotype in low-grade cancer to an invasive phenotype in higher-grades may explain why genes (*COL1A2* and *SPARC*) coding for extracellular matrix (ECM) proteins are independently upregulated in higher-grade stroma but not in low-grade stroma. Cross-linking of CAF-secreted collagens has been shown to promote tumor invasion by increasing ECM stiffness [65]. Binding of secreted COL1A2 to integrins, such as integrin *α*2*β*1 on the surface of tumors, has also been shown to drive tumor cell migration via the activation of RhoC and PI3 kinase [66]. SPARC has been shown to promote type 1 collagen fibril accumulation and remodelling and to facilitate migration of CAFs by decreasing adhesion to the ECM [67]. Interestingly, SPARC may also play an additional role in controlling metabolism. Its overexpression in the epithelial compartment has been shown to decrease glucose uptake and lactate production [68].

Finally, based on the protein-protein network analysis presented here, we propose that the reduction in RWE that we have observed occurs through the mediation of FOXO1 function. FOXO1 is controlled by competing pathways in the cell, namely activation by ROS [69–71], and inhibition via the insulin-activated PI3K/AKT pathway [72]. Decreased expression of *ATG5* in high-grade stroma is consistent with the effects of an activated PI3K/AKT1 pathway. The tendency of GP4 foci to overexpress growth signals such as IGF and EGFR may serve to explain FOXO1 activation in response to GP3 but not GP4 [73]. These results further illustrate the relationship between PCa phenotype and stromal metabolic response.

## Conclusions

Few studies have investigated the use of RWE-associated genes in a prognostic setting. In this study, we have identified two potential classifiers, stromal *FOXO1* and stromal *ATG5/GLUT1*, which have the potential to be used to distinguish between aggressive and indolent forms of PCa. Future directions should include validation of either *FOXO1* expression or *ATG5/GLUT1* relative expression classifiers in a larger cohort of independent tissue samples. Additionally, we report reduced expression of an RWE gene signature in high-grade stroma, a finding sufficiently robust to achieve significance under two independent statistical analyses. Based on the pathway analysis presented here, this reduction is likely mediated by FOXO1 and AKT1 signalling. Since the role that the RWE and its associated functional pathways play in PCa growth has to our knowledge not been previously explored, the work reported here indicates the potential benefits of investigating, measuring, and manipulating this pathway in PCa.

## Additional files

Additional file 1 Clinical data. **Table S1.** Correlations between GP3 and GP4 and secondary clinical characteristics: age, pre-op PSA, % cancer tissue volume, and pathological stage. (PDF 43.5 kb)

Additional file 2 Gene/accession numbers. **Table S2.** RWE code-set accession numbers. (XLSX 46.8 kb)

Additional file 3 MTE primer sequences. **Table S3.** MTE primer sequences. (XLSX 45.9 kb)

Additional file 4 NanoString probe sequences. **Table S4.** NanoSNanoStringtring probe sequences. (XLSX 42.1 kb)

Additional file 5 NanoString positive controls. **Figure S1.** Plot of the log_2_ raw expression values for six positive controls ranging from a concentration of 128 to 0.128 fM. Each box-whisker construct represents one positive control. The settings for the notched box-whisker plots are the same as those in the main text (see caption to Fig. 1). (PDF 5.09 kb)

Additional file 6 NanoString negative controls. **Figure S2.** Plot of the log_2_ raw expression values for eight negative controls. The broad distribution is likely due to the fragmentation of small input samples. The broad distribution of the negative controls indicates that the low intensity data is going to be less reliable. Each box-whisker construct represents one negative control. The settings for the notched box-whisker plots are the same as those in the main text (see caption to Fig. 1). (PDF 5.52 kb)

Additional file 7 NanoString housekeeping genes. **Figure S3.** Plot of the log_2_ raw expression values for the five selected housekeeping genes. Distribution of expression values is very broad within each gene; *HPRT1* and *TUBB* have particularly long whisker ranges of 5000. The very broad distribution of these housekeeping genes in stromal tissue made them unamendable to the calculation of normalization factors; therefore normalization factors were calculated using the geometric mean of the top 75 genes within a sample. The settings for the notched box-whisker plots are the same as those in the main text (see caption to Fig. 1). (PDF 5.02 kb)

## Abbreviations

AUC: area under the curve
CAF: cancer-associated fibroblast
ECM: extracellular matrix
FDR: false discovery rate
FFPE: formalim-fixed paraffin-embedded
FOV: field of view
GO: Gene Ontology
GP: Gleason pattern
GS: Gleason score
MTE: multiplex target enrichment
MWU: Mann-Whitney *U*
PCa: prostate cancer
ROC: receiver operator characteristics
ROS: reactive oxygen species
TSP: top-scoring pair
