# Supplementary material for: Molecular characterization of Gleason patterns 3 and 4 prostate cancer using reverse Warburg effect-associated genes
Source: Cancer Metab. 2016 May 5;4:8. doi: 10.1186/s40170-016-0149-5 (PMC4857335; doi:10.1186/s40170-016-0149-5)
Supplement: Additional file 1 — Clinical data. Table S1. Correlations between GP3 and GP4 and secondary clinical characteristics: age, pre-op PSA, % cancer tissue volume, and pathological stage. (PDF 43.5 kb) [file 40170_2016_149_MOESM1_ESM.pdf]

	Gleason Pattern		
	**GP4 (N=19)**	**GP3 (N=15)**	P-value
**Age (n=34)**	61.4 (6.0)	56.3 (7.4)	0.03
**Pre-op PSA (n=34)**	9.2 (6.4)	5.3 (2.4)	0.01
**% Cancer Tissue (n=34)**	50.3 (28.5)	31.3 (25.8)	0.05
**T stage (Pathological) (n=29)**			0.01
T1a-T1c	1 (6.3%)	3 (23.1%)	
T2a-T2c	9 (56.3%)	10 (76.9%)	
T3a-T3b	6 (37.4%)	0 (0.0%)	

For categorical variables used Mantel-Haenszel test
For continuous variables used T test
