# Supplementary figures and images for: Molecular characterization of Gleason patterns 3 and 4 prostate cancer using reverse Warburg effect-associated genes
Source: Cancer Metab. 2016 May 5;4:8. doi: 10.1186/s40170-016-0149-5 (PMC4857335; doi:10.1186/s40170-016-0149-5)

## RWE – Positive Controls

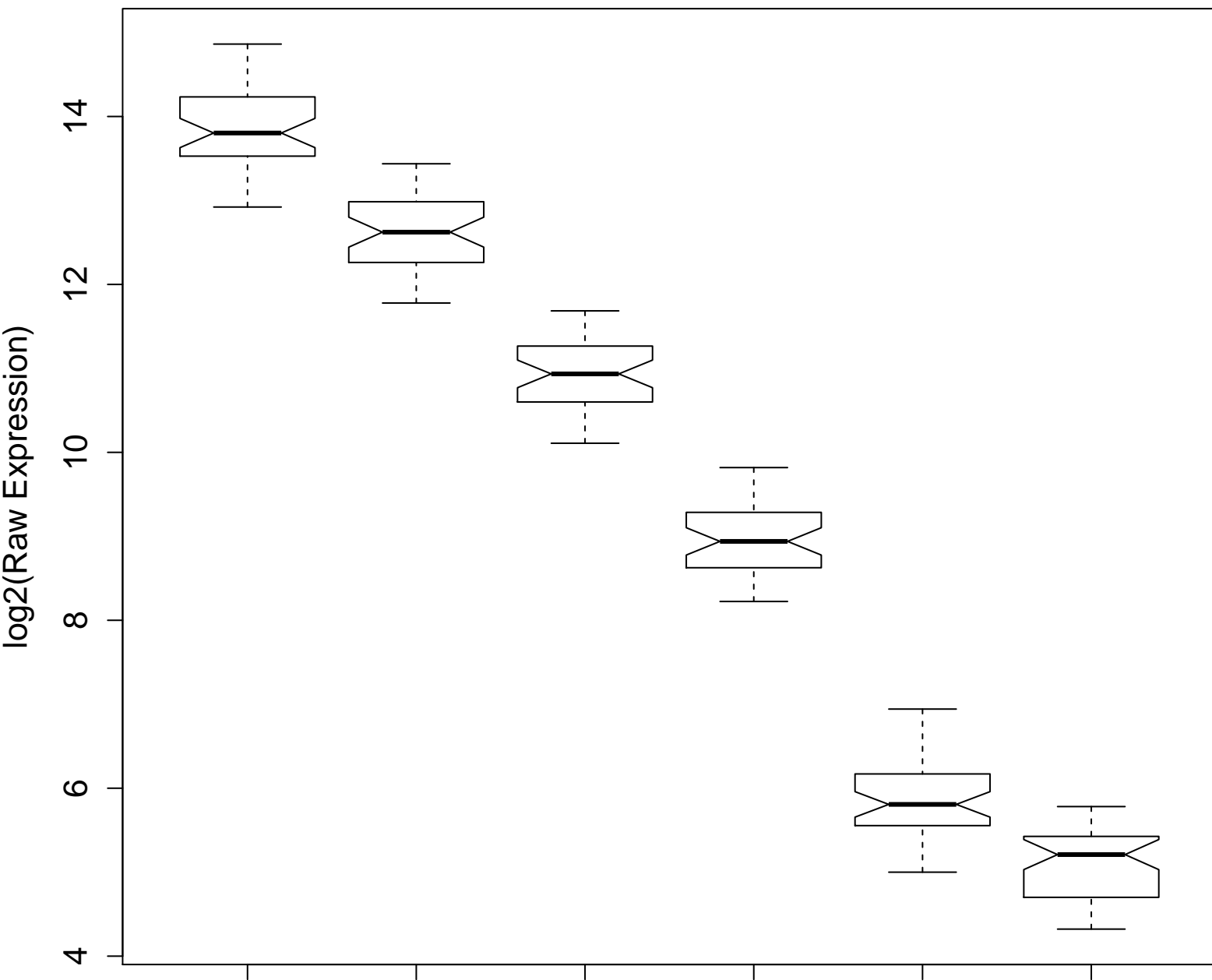

Supplement: Additional file 5 — NanoString positive controls. Figure S1. Plot of the log2 raw expression values for six positive controls ranging from a concentration of 128 to 0.128 fM. Each box-whisker construct represents one positive control. The settings for the notched box-whisker plots are the same as those in the main text (see caption to Fig. 1). (PDF 5.09 kb) [file 40170_2016_149_MOESM5_ESM.pdf]

# RWE – Negative Controls

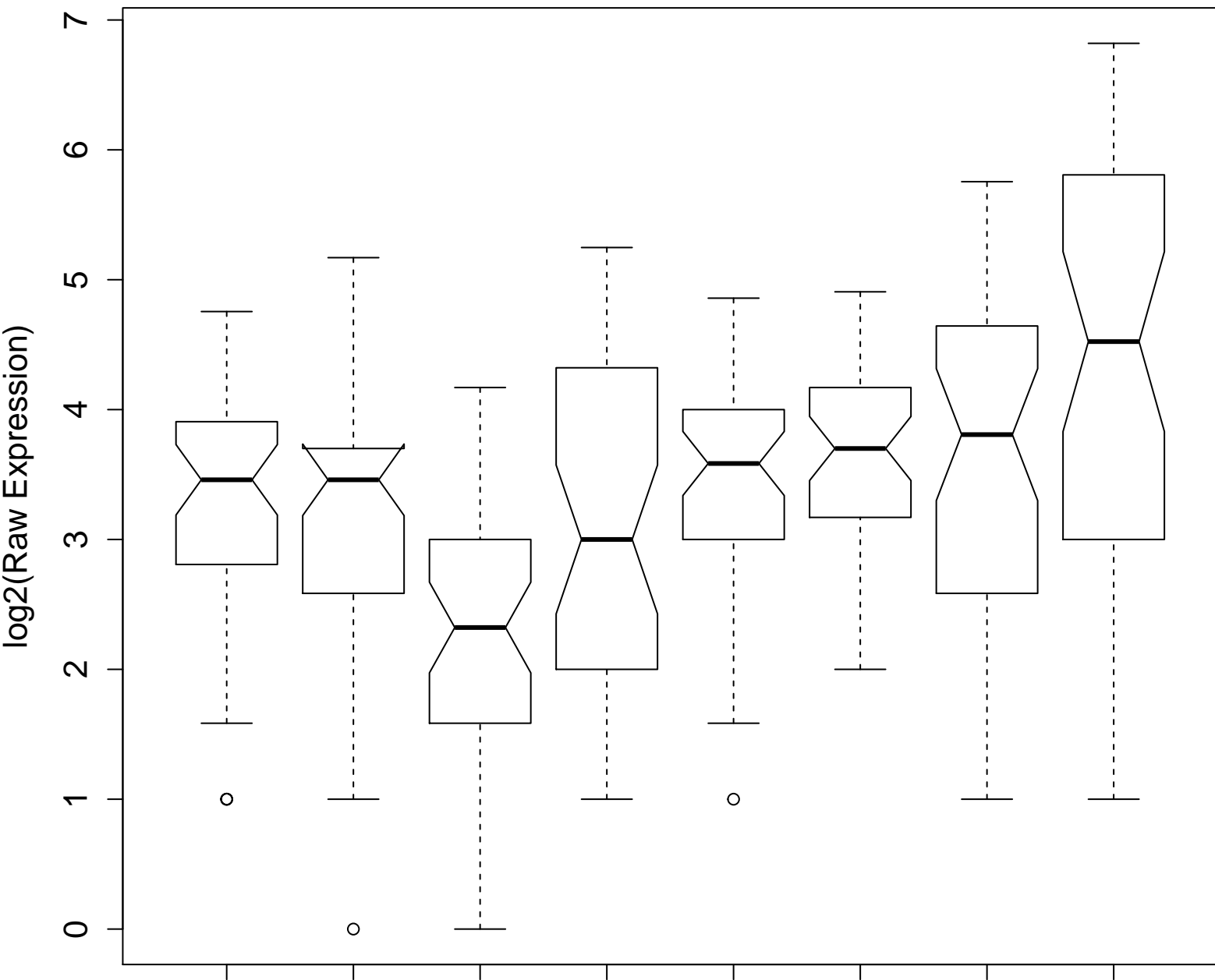

Supplement: Additional file 6 — NanoString negative controls. Figure S2. Plot of the log2 raw expression values for eight negative controls. The broad distribution is likely due to the fragmentation of small input samples. The broad distribution of the negative controls indicates that the low intensity data is going to be less reliable. Each box-whisker construct represents one negative control. The settings for the notched box-whisker plots are the same as those in the main text (see caption to Fig. 1). (PDF 5.52 kb) [file 40170_2016_149_MOESM6_ESM.pdf]

# RWE – HK Genes

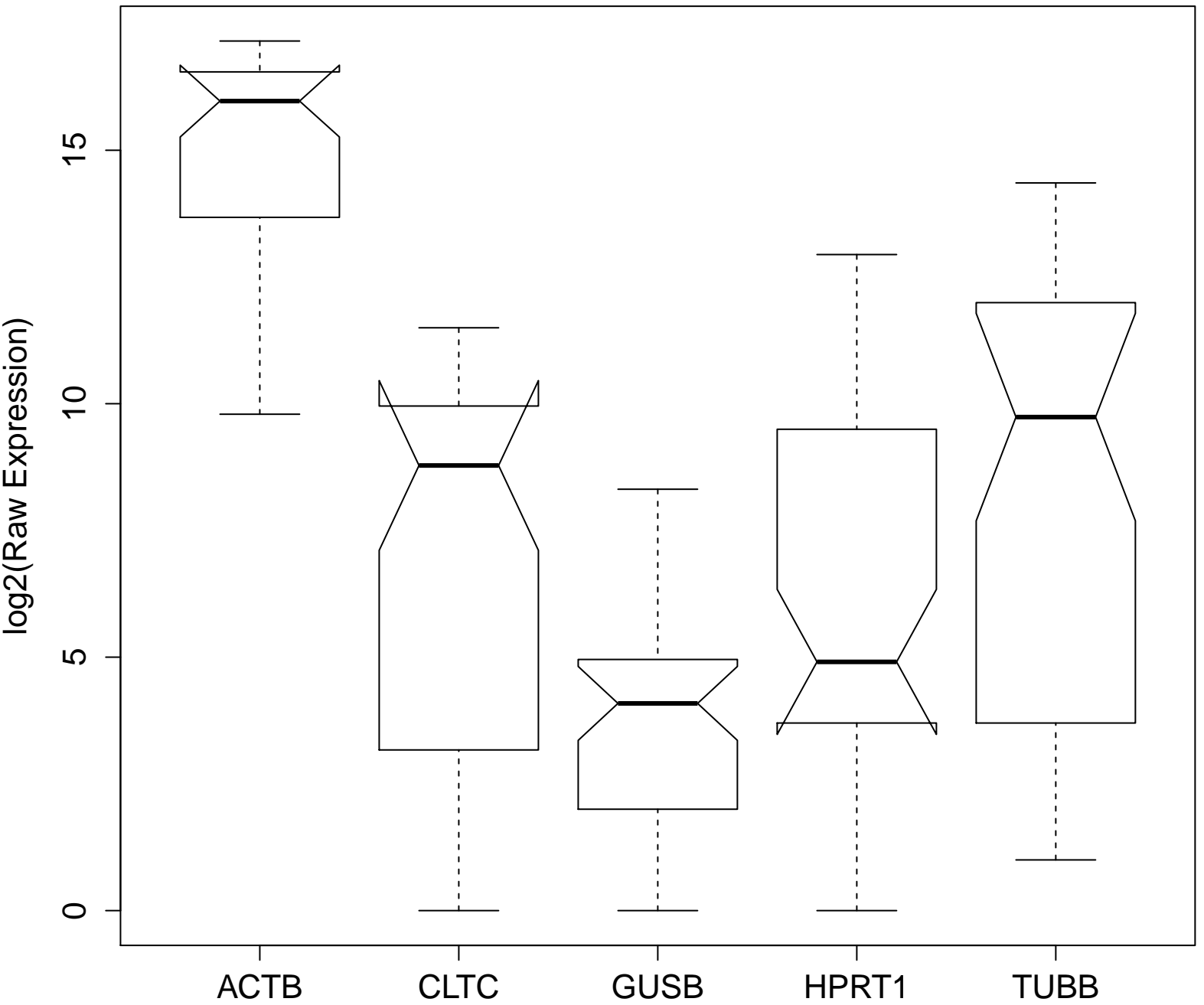

Supplement: Additional file 7 — NanoString housekeeping genes. Figure S3. Plot of the log2 raw expression values for the five selected housekeeping genes. Distribution of expression values is very broad within each gene; HPRT1 and TUBB have particularly long whisker ranges of 5000. The very broad distribution of these housekeeping genes in stromal tissue made them unamendable to the calculation of normalization factors; therefore normalization factors were calculated using the geometric mean of the top 75 genes within a sample. The settings for the notched box-whisker plots are the same as those in the main text (see caption to Fig. 1). (PDF 5.02 kb) [file 40170_2016_149_MOESM7_ESM.pdf]
